# Achieving Gender Equality Requires Placing Adolescents at the Center

**DOI:** 10.1016/j.jadohealth.2019.02.002

**Published:** 2019-06

**Authors:** Robert Wm Blum, Jo Boyden, Annabel Erulkar, Caroline Kabiru, Siswanto Wilopo

**Affiliations:** a Johns Hopkins Bloomberg School of Public Health, Baltimore, Maryland; b University of Oxford, Oxford, United Kingdom; c Population Council, Addis Ababa, Ethiopia; d Population Council, Nairobi, Kenya; e Gadjah Mada University, Yogyakarta, Indonesia

Today, there are 1.8 billion young people aged 10–24 years or one quarter of the world's population, 86% of whom live in low- and middle-income countries [1]. In many ways, this is good news—one of the greatest legacies of the Millennium Development Goals is the reduction in infant and child mortality worldwide from 12.7 per 1,000 in 1990 to 5.9 in 2015 [2]. During that same period, child survival was not matched, however, with declining birth rates; so too, investments in the first 1,000 were not matched with investments in the first 8,000 days of life [3]. As a consequence, large segments of the global population of young people have not been beneficiaries of global economic growth, and now large segments of this same population face steep challenges that threaten its developmental and economic potential. In many corners of the world, crushing poverty and a dearth of opportunity constrain both individual and national development, adversely impacting the prospects of adolescents more than any other age group. Going forward, as fertility rates decrease, and a country's working-age population grows relative to its dependent population, there is a window of opportunity for rapid economic growth if the right social and economic investments and policies are made in health, education, governance, and the economy [3]. However, realizing this dividend will require that all segments and genders of the population to be equal beneficiaries.

No longer can countries afford to discount the contributions of half their population by restricting opportunities and access for girls and women. Neither can they afford to ignore the personal and social consequences of gender discrimination. The United Nations reflected this imperative in the Sustainable Development Goals (SDGs) where gender equality and empowerment of all girls and women are one of the pillars (SDG#5). So too, The World Bank has indicated that gender inequalities and gender norms that entrench gender discrimination impact not only individuals but are also perhaps the biggest constraints on a country's economic growth [4]. However, if SDG #5 is to be achieved by 2030, we cannot ignore boys and men in the name of supporting girls and women. Neither can we wait for adulthood to increase social inclusion and produce generational change.

## Why Early Adolescence Aged 10–14 Years?

If we are to achieve gender equality, we must start our programs and policies before gender discriminatory norms become entrenched and the associated problems become refractory [5]. Puberty is a critical period of both opportunity and risk, especially for those living in the poorest communities [6]. With the onset of puberty, which is occurring earlier worldwide [7], there is emergence of sexual awareness, sexual identity, and gender role formation. Time use data from the Young Lives study in Ethiopia, for example, suggest that from around age 12 years, girls become more involved with unpaid domestic work, whereas boys spend increasing time in productive work that has career potential. Ages 12–15 years are crucial in this labor socialization process, and by 16 or 17 years, these roles become more solidified [8]. Likewise, in low-income communities, the social worlds of pubescent girls shrink while that of boys increase (see, e.g., the CRISP Trust study of spatial mapping for pre- and post-pubescent boys and girls) [9]. The gender intensification hypothesis suggests that early adolescence is a window of opportunity before norms and behaviors become established patterns for the next generation [10].

## Gender Inequalities and Their Consequences in Adolescence

There is a prevailing myth that girls alone are disadvantaged by unequal gender norms. The data suggest otherwise. Specifically, the evidence is that both boys and girls are disadvantaged, some in similar and some in unique ways. The Global Early Adolescent Study pilot data suggest that boys experience as much disadvantage as girls (e.g., fear of being physically hurt, neglected, a victim of sexual or physical violence) [11]. So too, public health data indicate that in many countries of the world, adolescent boys are more likely to smoke, drink, and suffer both unintentional and intentional injury and death in the second decade of life than their female counterparts [12]. Conversely, one quarter of adolescent girls worldwide are married by age 18 years, two million births annually are to girls aged younger than 15 years, and girls' secondary school education still lags behind boys (56%–63%) [13]. Social and vocational opportunities are frequently more constrained for girls than their male counterparts.

But these statistics tell only part of the story for while the data are cast in terms of gender disadvantage, that disadvantage is not equally distributed across the socioeconomic spectrum; rather, in low- and high-income countries alike, those in the poorest quintile are more likely to leave school earlier, have children earlier, and marry earlier while still in childhood than their higher income peers. Poverty and gender inequality together conspire to disadvantage large segments of the adolescent population.

## What Needs to Be Done to Achieve Gender Equality

In May 2018, a group of 22 global leaders (The Bellagio Working Group) met for 3 days to craft indicators and research priorities that can inform programs and policies aimed at achieving gender equality for all by 2030 (SDG#5).

The central recommendations to improve policy and practice include:

1. Place adolescents at the center of strategies that increase gender equality and start with early adolescence.
2. Acknowledge that there are multiple structural factors that drive gender disparities; thus, individual behavior change and empowerment approaches alone are insufficient.
3. Re-examine the roles that education and faith institutions play in enforcing gender inequalities and develop institutional strategies to maximize gender equality and inclusion.
4. Expand SDG indicators so as to prioritize and better monitor changes needed to maximize gender equality. These indicators focus on key aspects of adolescents' lives where the greatest changes are needed, especially education, health, personal safety, and empowerment. Indicators need to include the percentage of adolescents:
  a. *Education*: Completing secondary school by sex, with access to comprehensive sex education.
  b. *Freedom from violence:* Feeling safe in their neighborhood and school by age and sex, exposed to gender-based and interpersonal violence by age and sex.
  c. *Freedom from coercion:* Marrying by age 15 and 18 years.
  d. *Voice:* Indicating they can ask for help when needed.
  e. *Health:* Reporting depression by age and sex, accessing family planning.
5. Research priorities: Based on discussions of the critical evidence gaps and needs, the Bellagio Working Group highlighted that research is needed to enhance understanding in the following areas:
  a. Impact evaluation of laws related to child marriage, female genital cutting, gender-based violence, universal secondary education.
  b. Improved estimates of gender-based violence (perpetration and victimization) by sex.
  c. Deeper understanding of the social contexts and structural factors impacting gender equality for low-income adolescents.
  d. Consequences of excessive and/or nonproductive unpaid work during early adolescence for subsequent education, marriage, and employment.
  e. Replication and scale-up of effective programs that increase gender equality.
  f. Improved measures of quality assessment for adolescent health service delivery and sex education.
  g. Expanded impact evaluation of gender norm change and other interventions aimed at increasing gender equality.
6. An international agency or nongovernmental organizations should be tasked and funded to monitor the proposed adolescent indicators for SDG #5. Without a deliberate monitoring and reporting system in place, achievement of the adolescent indicators for SGD #5 is less likely.

To achieve gender equality for all by 2030 (SDG#5), adolescents must be seen as central, for it is they who are most impacted by gender inequalities over time, and it is they who hold the promise of reversing current inequalities. The Bellagio Working Group recognized that a number of existing indicators both within SDG#5 and other SDGs warrant more effective monitoring. But the Group also proposed that additional indicators and research priorities are needed to ensure movement toward gender equality for adolescents over the next 12 years.

To achieve gender equality, we need to redefine the problem as a “gender,” not women's and girls', issue. And the evidence is strong that the “…links between gender equality and life satisfaction among adults suggest that men as well as women benefit from high level of societal gender equality. Moreover, an abundance of literature shows that males as well as females benefit from a more socially supportive climate” [14]. The time to act is now, for with the SDGs, there is opportunity to focus global attention on the priority of adolescents in achieving gender equality.
